# A case of laparoscopic lymphadenectomy for adenocarcinoma of unknown primary incidentally detected as a solitary enlarged lymph node along the common hepatic artery

**DOI:** 10.1186/s40792-024-01888-9

**Published:** 2024-04-18

**Authors:** Tomonori Morimoto, Shigeo Hisamori, Hiromitsu Kinoshita, Yosuke Yamada, Yuki Teramoto, Takashi Sakamoto, Keiko Kasahara, Shintaro Okumura, Tatsuto Nishigori, Shigeru Tsunoda, Kazutaka Obama

**Affiliations:** 1 Department of Gastrointestinal Surgery, Kyoto University Hospital, 54 Shogoin-Kawahara-Cho, Sakyo-Ku, Kyoto, 606-8507 Japan; 2 Department of Diagnostic Pathology, Kyoto University Hospital, 54 Shogoin-Kawahara-Cho, Sakyo-Ku, Kyoto, 606-8507 Japan

**Keywords:** Cancer of unknown primary, Solitary lymph node metastasis, No.8a lymph node, Laparoscopic resection

## Abstract

**Background:**

Even in cancer of unknown primary (CUP), which is rare clinical condition, solitary anterosuperior lymph node (LN) along the common hepatic artery (No.8a LN) enlargement diagnosed as metastatic adenocarcinoma has never been reported.

**Case presentation:**

A 68-year-old Japanese male, with a history of early gastric cancer that had been completely treated by endoscopic submucosal dissection 26 years ago, was detected a single enlarged nodule along the common hepatic artery, No.8a LN, incidentally by computed tomography performed for monitoring of interstitial pneumonia. Endoscopic ultra-sound-guided fine needle aspiration revealed that this nodule was adenocarcinoma suggestive of metastasis, but other imaging studies, including upper and lower gastrointestinal endoscopy, positron emission tomography, and ultrasonography did not detect any primary cancer. We have finally diagnosed as the LN metastasis of CUP and performed laparoscopic lymphadenectomy for this tumor. The tumor was approximately 5 cm in size, was in close proximity to the pancreas, and involved part of the right gastric artery and vein. LNs in the No.5 and No.8a areas, including this tumor, were dissected laparoscopically, and radical resection was achieved. The patient had no postoperative complication and was discharged on postoperative day 10. Immunohistopathological findings revealed that the tumor was poorly differentiated adenocarcinoma, and different from the histology of gastric cancer resected 26 years ago, although the tumor was suggestive of gastrointestinal origin. Imaging studies performed 2 and 6 months after discharge also did not reveal a primary site.

**Conclusion:**

We reported a case of solitary No.8a LN adenocarcinoma of CUP. For diagnostic and therapeutic purposes, radical resection is recommended for single enlarged intra-abdominal LN of CUP.

## Background

The cancer unknown primary (CUP) is a well-recognized clinical condition, accounting for 3–5% of all malignant cancer [1–3]. The histological classification showed adenocarcinoma in about 60% cases of CUP, and the half number of metastatic site was reported to be in the lymph nodes (LNs); cervical, mediastinal, and retroperitoneal LNs are major cite [4, 5]. On the other hand, intra-abdominal LN adenocarcinoma is 1–2% of CUP, and solitary anterosuperior LN along the common hepatic artery (CHA; No.8a LN) of CUP has not been reported. Stereotypical treatment of these metastatic LN is same as lymphadenectomy associated with gastrectomy [6], and minimal invasive surgery is becoming standard procedure [7].

In this report, we described the case of single LN enlargement along the CHA diagnosed as metastatic adenocarcinoma with CUP, having history of completely treated early gastric cancer (GC), and underwent laparoscopic radical LN dissection successfully.

## Case presentation

**Fig. 1:**
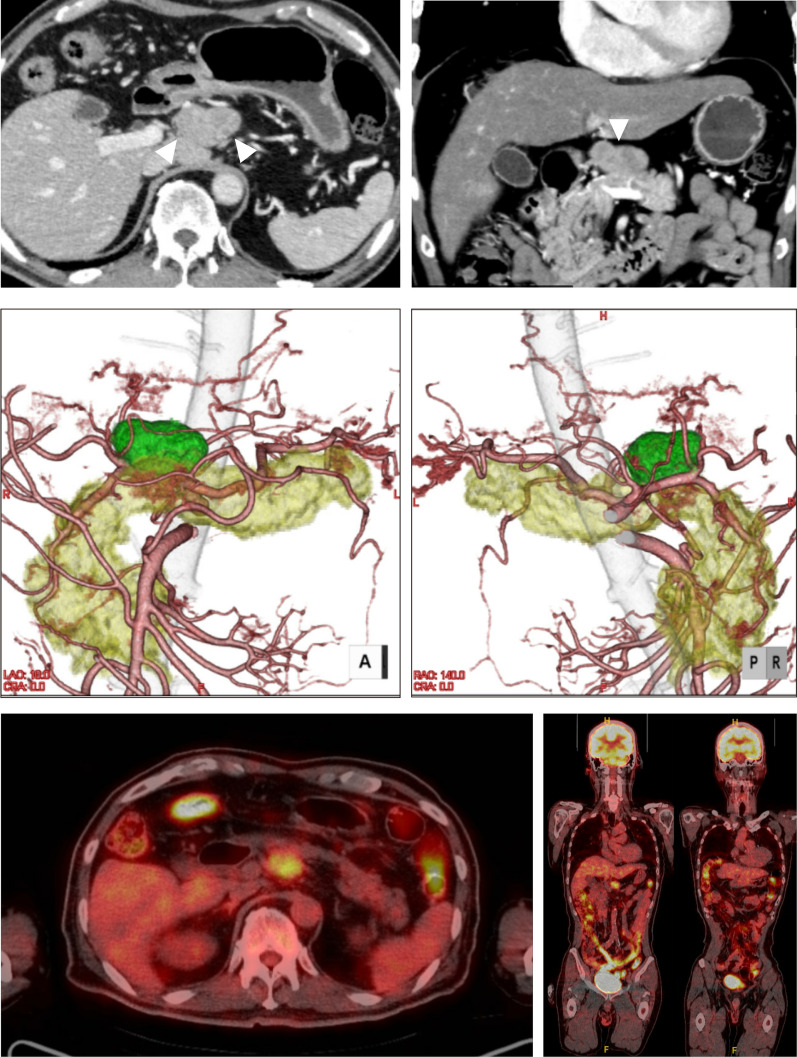
The preoperative contrast-enhanced CT and FDG-PET images. These images showed metastatic tumor in No.8a LN (top), 3D images containing vascular reconstruction (middle), and FDG-PET CT images of No.8a LN (bottom, left) and whole body (bottom, right). Arrowheads indicated the tumor contact with pancreas and close proximity to right gastric artery

The patient was a 68-year-old Japanese male, being followed up for interstitial pneumonia (IP) and got medication of nintedanib. He had undergone endoscopic submucosal dissection (ESD) for GC in another hospital 26 years ago. That tumor had been located in antrum, 5 × 5 mm in size, limited invasion into mucosa (T1a), and no lymphovascular invasion. Follow-up surveillance for that GC had been finished. He had other history of diabetes mellites, urinary stone, and chronic urticaria, and he got medication of omalizumab for chronic urticaria. At the time of routine follow-up for IP, computed tomography (CT) revealed a single swelling nodule in the area of No.8a LN. This swelling had not observed six months earlier. Positron emission tomography (PET) showed that 18F-fluorodeoxyglucose (FDG) was taken up by this nodule (SUV max: 4.2), and it was suggested to be a malignant tumor. There was no nodule taking FDG except this tumor. The tumor was in contact with pancreas and close proximity to right gastric artery or CHA (Fig. 1). Endoscopic ultra-sound (EUS) showed no cancer site in pancreas or bile duct, and EUS guided fine needle aspiration revealed that this nodule was adenocarcinoma suggestive of metastasis from gastrointestinal tract, but multiple diagnostic modalities, including CT, FDG-PET, ultrasonography, upper and lower gastrointestinal endoscopy did not detect any primary cancer. The level of cancer marker CEA and CA19-9 was not elevated. The level of CA125 was elevated (47.3 U/ml), but elevation of CA125 had been observed since the initial visit of IP, a year before resection. We concluded that this tumor might be the metastasis of GC 26 years ago and decided to perform radical resection of this tumor for accurate diagnosis and curative treatment as well.

**Fig. 2:**
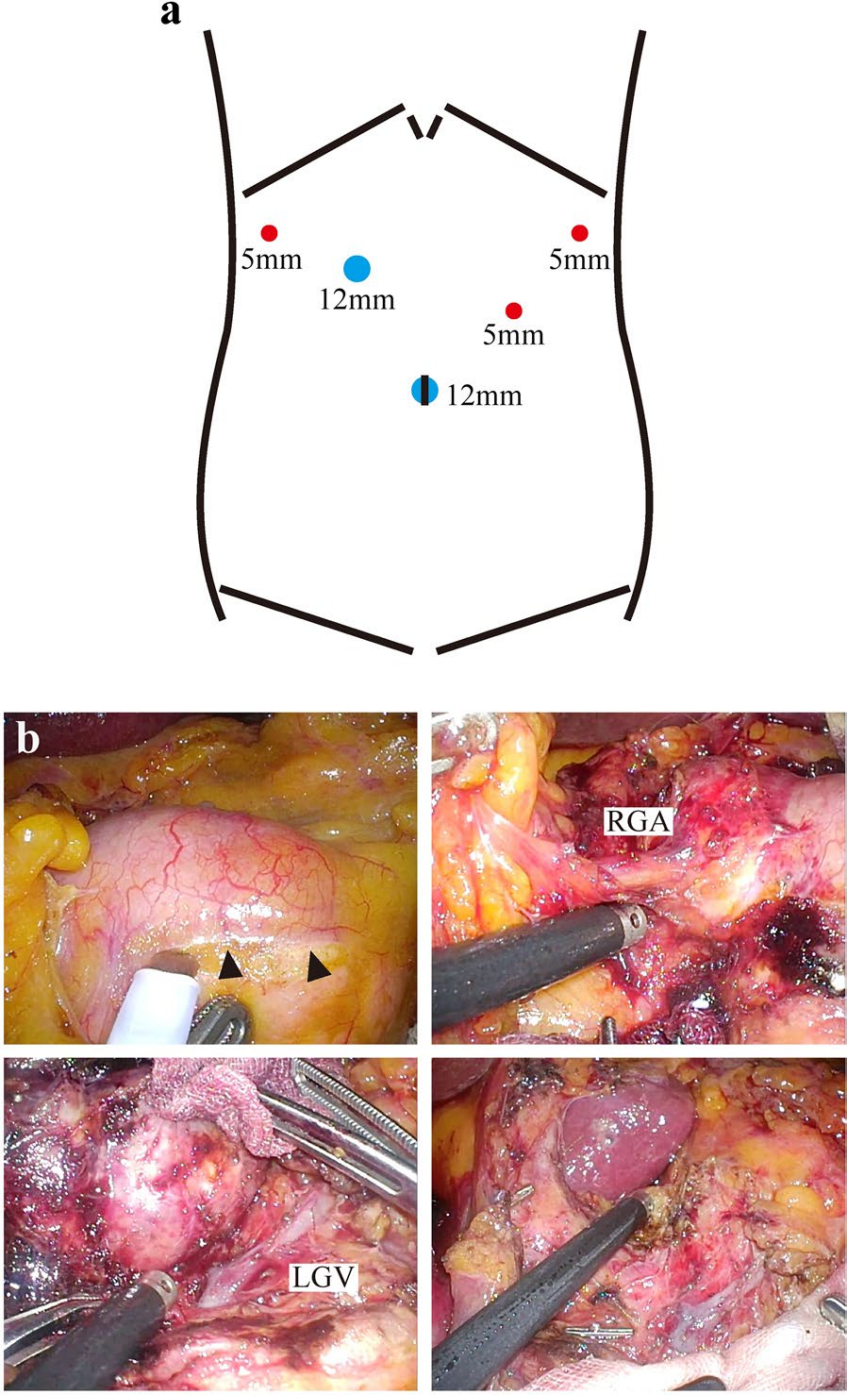
Operation findings. **a** Trocar placement. We placed 5 ports (two 12-mm ports and three 5-mm ports). **b** Tumor was in contact with pancreas. Arrowheads showed the border of tumor and pancreas (top, left). Right gastric artery was involved into tumor (top, right), but left gastric vein was released from it (bottom, left). Tumor was resected as the way of suprapancreatic LN dissection (bottom, right). RGA: right gastric artery; LGV: left gastric vein

We performed metastatic LN resection laparoscopically. Five ports were placed according to the standard laparoscopic distal gastrectomy, but arranged the 12-mm port on the surgeon’s right hand headward than usual (Fig. 2a). The intraoperative findings showed that the tumor was contact with pancreas, and it involved both right gastric artery and vein (RGAV) (Fig. 2b). We carefully separated the tumor from pancreas, and dissected RGAV. The tumor was dissected successfully with preserving the left gastric artery and vein, taking care not to damage the tumor capsule. No other swelling LN was detected during the operation. Operation time was 4 h and 13 min, and the amount of blood loss was very minimal. Amylase level of drainage fluid was not elevated in postoperative day (POD) 1. The patient was discharged on POD 10 without any postoperative complication.

**Fig. 3:**
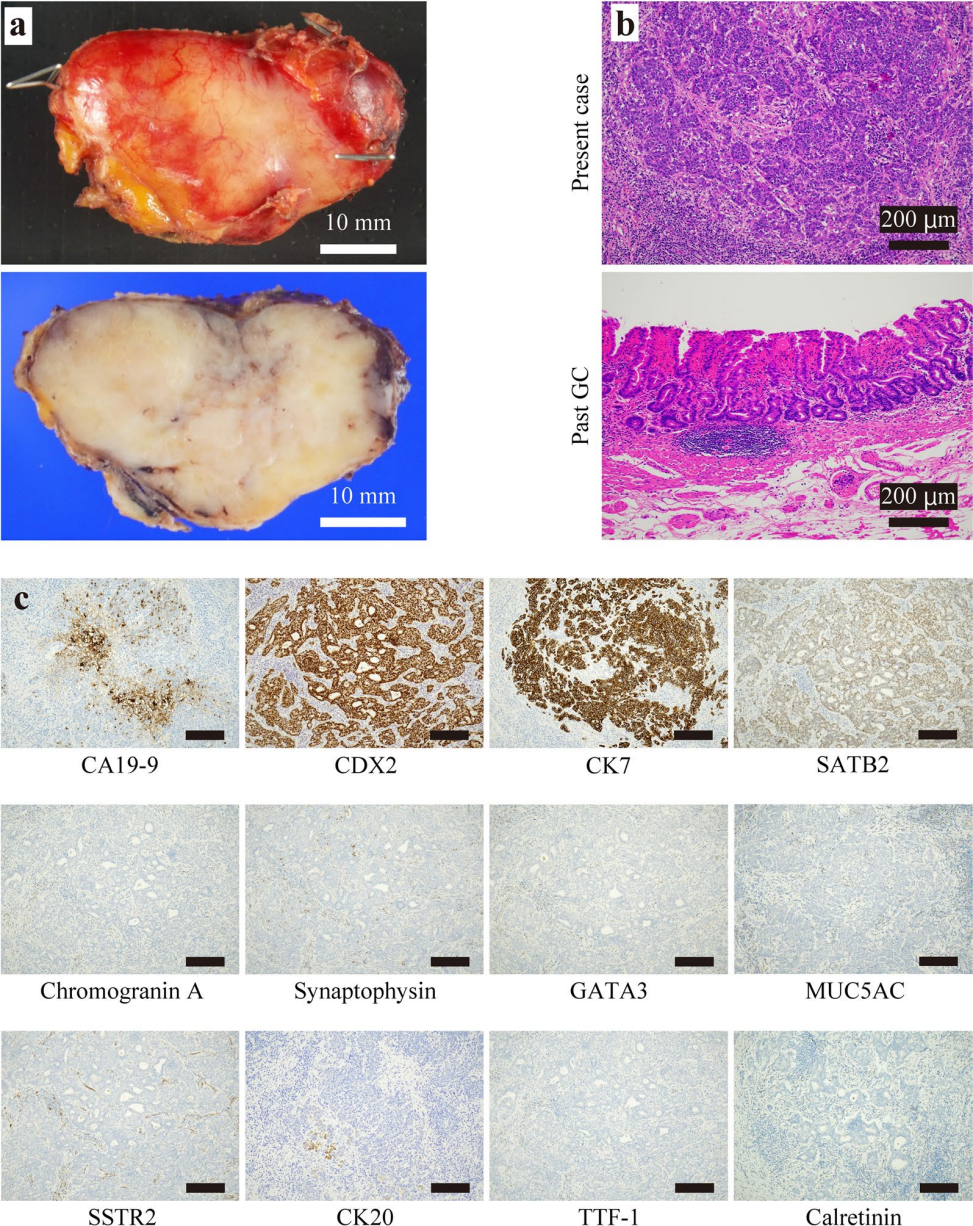
Image of resected tumor. **a** Macroscopic (top) view and cross-sectional view (bottom) of the resected tumor. **b** Microscopic view of hematoxylin–eosin-stained sections from resected tumor (present case, top) and specimen from GC resected endoscopically 26 years ago (past GC, bottom). **c** Immunohistochemistry for markers of resected tumor. Scale bar; 200 μm

The histopathological findings indicated that the tumor was 40 × 35 mm in size, encapsulated LN that was composed of poorly differentiated adenocarcinoma cells potentially originated from gastrointestinal cells (Fig. 3a, b). Immunostaining was positive for carbohydrate antigen 19-9 (CA19-9), caudal-related homeobox transcription factor 2 (CDX2), cytokeratin 7 (CK7), and special AT-rich sequence-binding protein 2 (SATB2), negative for chromogranin A, synaptophysin, GATA binding protein 3 (GATA3), mucin 5AC (MUC5AC), somatostatin receptor 2 (SSTR2), cytokeratin 20 (CK20), thyroid transcription factor-1 (TTF-1), and calretinin (Table 1, and Fig. 3c). A specimen of GC resected 26 years ago was retrieved from another hospital and examined for histology, and found to be well-differentiated adenocarcinoma, which was clearly different from the histology of the LN in the present case (Fig. 3b). Therefore, the single enlarged LN along the CHA was diagnosed as CUP. Upper gastrointestinal endoscopy and CT, which was performed 2 and 6 months after discharge, did not show any malignant findings including the recurrence.

**Table 1:** Summary of immunohistochemistry analysis of markers of cancer

Positive		Negative	
CA19-9	3%	Chromogranin A	
CDX2	80%	Synaptophysin	
CK7	70%	GATA3	
SATB2	50%	MUC5AC	
		SSTR2	
		CK20	1%
		TTF-1	
		Calretinin	

CA19-9: carbohydrate antigen 19–9; CDX2: caudal-related homeobox transcription factor 2; CK7: cytokeratin 7; SATB2: special AT-rich sequence-binding protein 2; GATA3: GATA binding protein 3; MUC5AC: mucin 5AC; SSTR2: somatostatin receptor 2; CK20: cytokeratin 20; TTF-1: thyroid transcription factor-1

## Discussion

This is the first report of solitary metastatic adenocarcinoma of CUP detected at the No.8a LN region. There have been three reports of perigastric or suprapancreatic LN metastasis derived from CUP, including squamous cell carcinoma, neuroendocrine tumor, and adenocarcinoma [8–10]. With regard to “No.8a LN” metastasis of adenocarcinoma of CUP, there was a case report of No.8a and No.3 metastasis [11], but the metastasis solitary in No.8a LN has never been reported. Furthermore, poorly differentiated abdominal LN metastasis of CUP is rare, less than well or moderately differentiated adenocarcinoma, neuroendocrine tumor, and squamous cell carcinoma [4]. Therefore, this case report is considered valuable because it suggests an extremely rare clinical condition that has never been discussed before.

We initially suspected that this tumor might be a recurrence from GC that had been treated by ESD 26 years ago. However, past GC was early stage, well-differentiated carcinoma, with no lymphovascular invasion, completely dissected, and recurrence has never observed for more than 20 years after resection. Moon et al. reported that rate of recurrence of advanced GC within 5–10 years was 8.8%, after 10 years was 2.0% [12], but in the case of early GC, no case report of recurrence has been found after more than 20 years. Furthermore, in terms of morphology, present tumor (poorly differentiated) and past GC (well-differentiated) was different. Wang et al. reported that there had been difference between preponderant histology of primary GC and histology of metastatic GC in 14.1% of cases [13], but these differences had been inferred to be due to heterogeneity of primary GC. The GC resected 26 years ago was consist of only well-differentiated adenocarcinoma, and expected not to be containing poorly differentiated adenocarcinoma. According to these results and reports, we concluded this tumor was not derived from the GC resected 26 years ago.

Immunohistochemistry findings of this case indicated that this tumor might be derived from gastrointestinal tract, mainly colon according to the NCCN guideline and ESMO guideline [3, 14], but no primary cancer was found in the abdomen or mediastinum on any of the examinations performed before or after the radical surgery. From these results, we finally concluded this LN metastasis was derived from CUP. For certain primary cancers, such as melanoma or ovarian cancer, immunohistochemistry of tumor marker is effective for diagnosis of primary regions [3, 14–17]. For gastrointestinal cancer, especially GC, however, there are few specific markers effective for diagnosis for primary regions, and it might be difficult to detect primary tumor of CUP by only histology or biological effort. Systemic and repetitive investigation (CT, upper and lower gastrointestinal endoscopy, FDG-PET CT) are essential to recognize the primary site [3, 14, 18].

Laparoscopic LN resection is standardized in laparoscopic gastrectomy, and suprapancreatic LN dissection is reported to be safe while resection is proceeded at the appropriate layer [19, 20]. In this case, tumor was in contact with pancreas, CHA and involved RGAV. However, by taking advantage of the magnification effect of laparoscopy and dissecting the layer bordering the pancreas and CHA, the tumor could be radically resected without damaging the tumor capsule. In these cases of adenocarcinoma of LN, we standardize the procedure of lymphadenectomy as the procedure of gastrectomy. Oncological and surgical safety of systemic gastrectomy contained lymphadenectomy in the case of LN metastasis is guaranteed by laparoscopic resection [7]. Laparoscopic lymphadenectomy for CUP can be considered safe to perform.

## Conclusion

We experienced a case of poorly differentiated adenocarcinoma of CUP detected as a solitary No.8a LN metastasis. Laparoscopic lymphadenectomy according to systematic LN dissection for gastrectomy could be performed safely. Continuous and systemic examination for surveillance of primary carcinoma and metastases is necessary after surgery.

## Data Availability

Data sharing is not applicable to this article, as no datasets were generated or analyzed.

## Abbreviations

CUP: Cancer unknown primary
LN: Lymph node
CHA: Common hepatic artery
No.8a LN: Anterosuperior lymph node along the common hepatic artery
GC: Gastric cancer
IP: Interstitial pneumonia
ESD: Endoscopic submucosal dissection
CT: Computed tomography
PET: Positron emission tomography
FDG: 18F-fluorodeoxyglucose
EUS: Endoscopic ultra-sound
RGAV: Right gastric artery and vein
POD: Postoperative day
CA19-9: Carbohydrate antigen 19-9
CDX2: Caudal-related homeobox transcription factor 2
CK7: Cytokeratin 7
SATB2: Special AT-rich sequence-binding protein 2
GATA3: GATA binding protein 3
MUC5AC: Mucin 5AC
SSTR2: Somatostatin receptor 2
CK20: Cytokeratin 20
TTF-1: Thyroid transcription factor-1

## References

[1] Pavlidis N, Fizazi K (2009). Carcinoma of unknown primary (CUP). Crit Rev Oncol Hematol.

[2] Pavlidis N, Pentheroudakis G (2012). Cancer of unknown primary site. Lancet.

[3] Krämer A, Bochtler T, Pauli C (2023). Cancer of unknown primary: ESMO clinical practice guideline for diagnosis, treatment and follow-up. Ann Oncol.

[4] Lenzi R, Hess KR, Abbruzzese MC (1997). Poorly differentiated carcinoma and poorly differentiated adenocarcinoma of unknown origin: favorable subsets of patients with unknown-primary carcinoma?. J Clin Oncol.

[5] Hemminki K, Bevier M, Hemminki A (2012). Survival in cancer of unknown primary site: population-based analysis by site and histology. Ann Oncol.

[6] Japanese Gastric Cancer Association (2023). Japanese gastric cancer treatment guidelines 2021 (6th edition). Gastric Cancer.

[7] Inaki N, Etoh T, Ohyama T (2015). A multi-institutional, prospective, phase II feasibility study of laparoscopy-assisted distal gastrectomy with D2 lymph node dissection for locally advanced gastric cancer (JLSSG0901). World J Surg.

[8] Lee HS, Han HS, Lim SN (2012). Poorly differentiated neuroendocrine carcinoma in a perigastric lymph node from an unknown primary site. Cancer Res Treat.

[9] Seshie BK, Kim KH, Lee HJ (2020). Synchronous gastric adenocarcinoma and perigastric lymph node metastatic squamous cell carcinoma with unknown primary: a case report. J Minim Invasive Surg.

[10] Yang H, He F, Xu W (2022). Clinical features of cancer with unknown primary site (clinical features, treatment, prognosis of cancer with unknown primary site). BMC Cancer.

[11] Ito E, Wakiyama S, Fujiwara Y (2014). A case of long-term survival without recurrence from an unknown primary carcinoma after resection of lymph node metastases. Nihon Shokakibyo Gakkai Zasshi.

[12] Moon YW, Jeung HC, Rha SY (2007). Changing patterns of prognosticators during 15-year follow-up of advanced gastric cancer after radical gastrectomy and adjuvant chemotherapy: a 15-year follow-up study at a single Korean institute. Ann Surg Oncol.

[13] Wang LB, Jiang ZN, Fan MY (2008). Changes of histology and expression of MMP-2 and nm23-H1 in primary and metastatic gastric cancer. World J Gastroenterol.

[14] Ettinger DS, Agulnik M, Cates JM (2011). NCCN clinical practice guidelines occult primary. J Natl Compr Canc Netw.

[15] Oien KA (2009). Pathologic evaluation of unknown primary cancer. Semin Oncol.

[16] Stella GM, Senetta R, Cassenti A (2012). Cancers of unknown primary origin: current perspectives and future therapeutic strategies. J Transl Med.

[17] Conner JR, Hornick JL (2015). Metastatic carcinoma of unknown primary: diagnostic approach using immunohistochemistry. Adv Anat Pathol.

[18] Ren M, Cai X, Jia L (2023). Comprehensive analysis of cancer of unknown primary and recommendation of a histological and immunohistochemical diagnostic strategy from China. BMC Cancer.

[19] Shinohara H, Kurahashi Y, Haruta S (2018). Universalization of the operative strategy by systematic mesogastric excision for stomach cancer with that for total mesorectal excision and complete mesocolic excision colorectal counterparts. Ann Gastroenterol Surg.

[20] Kumamoto T, Kurahashi Y, Niwa H (2020). Laparoscopic suprapancreatic lymph node dissection using a systematic mesogastric excision concept for gastric cancer. Ann Surg Oncol.

